# Effect of Antioxidant Supplementation on the Sperm Proteome of Idiopathic Infertile Men

**DOI:** 10.3390/antiox8100488

**Published:** 2019-10-16

**Authors:** Ashok Agarwal, Manesh Kumar Panner Selvam, Luna Samanta, Sarah C. Vij, Neel Parekh, Edmund Sabanegh, Nicholas N. Tadros, Mohamed Arafa, Rakesh Sharma

**Affiliations:** 1 American Center for Reproductive Medicine, Cleveland Clinic, Cleveland, OH 44195, USA; pannerm@ccf.org (M.K.P.S.); lsamanta@ravenshawuniversity.ac.in (L.S.); sharmar@ccf.org (R.S.); 2 Department of Urology, Cleveland Clinic, Cleveland, OH 44195, USA; VIJS@ccf.org (S.C.V.); PAREKHN3@ccf.org (N.P.); SABANEE@ccf.org (E.S.); 3 Redox Biology Laboratory, Department of Zoology, Ravenshaw University, Cuttack 753003, India; 4 Division of Urology, Southern Illinois University School of Medicine, Springfield, IL 62769, USA; nicktadros@gmail.com; 5 Department of Urology, Hamad Medical Corporation, Doha 00974, Qatar; mohamedmostafaarafa@gmail.com

**Keywords:** antioxidants, sperm proteome, idiopathic, male infertility, bioinformatics

## Abstract

Antioxidant supplementation in idiopathic male infertility has a beneficial effect on semen parameters. However, the molecular mechanism behind this effect has not been reported. The objective of this study was to evaluate the sperm proteome of idiopathic infertile men pre- and post-antioxidant supplementation. Idiopathic infertile men were provided with oral antioxidant supplementation once daily for a period of 6 months. Of the 379 differentially expressed proteins (DEPs) between pre- and post-antioxidant treatment patients, the majority of the proteins (*n* = 274) were overexpressed following antioxidant treatment. Bioinformatic analysis revealed the activation of oxidative phosphorylation pathway and upregulation of key proteins involved in spermatogenesis, sperm maturation, binding of sperm, fertilization and normal reproductive function. In addition, the transcriptional factors associated with antioxidant defense system (PPARGC1A) and free radical scavenging (NFE2L2) were predicted to be functionally activated post-treatment. Key DEPs, namely, NDUFS1, CCT3, PRKARA1 and SPA17 validated by Western blot showed significant overexpression post-treatment. Our novel proteomic findings suggest that antioxidant supplementation in idiopathic infertile men improves sperm function at the molecular level by modulating proteins involved in CREM signaling, mitochondrial function and protein oxidation. Further, activation of TRiC complex helped in nuclear compaction, maintenance of telomere length, flagella function, and expression of zona pellucida receptors for sperm–oocyte interaction.

## 1. Introduction

Despite advances in the field of male reproductive health, idiopathic male infertility remains a challenging condition to diagnose and manage. Idiopathic male infertility is defined as the presence of altered semen characteristics without an identifiable cause and the absence of female factor infertility [1]. Increasing evidence suggests that oxidative stress (OS) plays a pivotal role in the etiology of male infertility [2,3,4,5]. Approximately 30% to 80% of infertile men having elevated seminal reactive oxygen species (ROS) levels suffer from a condition known as male oxidative stress infertility (MOSI) [1] which impacts 37 million men with idiopathic infertility.

Increased OS has deleterious effects on semen parameters such as sperm concentration, motility and progressive motility [6,7] and it also induces sperm DNA fragmentation (SDF) [2,8,9,10] resulting in male infertility. Endogenous antioxidants act on excess seminal oxidants to reduce the OS. In idiopathic male infertility cases, antioxidant supplementation is one of the treatment choices. Several studies have reported the beneficial effect of antioxidants on semen parameters. Oral intake of individual antioxidants or a combination of several antioxidants such as l-carnitine, selenium, N-acetyl-cysteine, Coenzyme Q10, ubiquinol, vitamin E, vitamin C, and lycopene have led to improvement in semen parameters and sperm DNA integrity of idiopathic infertile men [11,12,13,14,15]. 

Recently, Arafa et al. investigated the effect of ‘FH PRO for Men’ antioxidant in 148 randomly selected idiopathic infertile men attending the clinic whose partners had failed to conceive for at least 12 months (mean infertility duration of 5.9 ± 4.2 years in the age group of 35.9 ± 0.5 years and body mass index of 29.6 ± 0.4 kg/m^2^). Post antioxidant treatment, the patients showed an 37.52% (*p* ≤ 0.05) increase in sperm concentration while total motility, progressive motility and normal morphology showed 11.22% (*p* ≤ 0.05), 101.50% (*p* ≤ 0.05) and 39.16% (*p* ≤ 0.05) augmentation, respectively. On the other hand, a 17.06% (*p* ≤ 0.05) and 39.47% (*p* ≤ 0.05) decline was observed for sperm DNA fragmentation and static oxidation-reduction potential (sORP). No complications/side effects were reported in any of the participants [16]. Likewise, a study by Gharagozloo et al. on an antioxidant supplement, Fertilix^®^ (combined antioxidant formulation) involving established mouse models of oxidative stress such as scrotal heat shock and GPx5 knockout mice have shown protection against oxidative DNA damage and antioxidant gene expression in cauda epididymal spermatozoa as well as pregnancy rate [17]. However, the molecular mechanism(s) behind such changes in human spermatozoa have not been reported.

Proteomics has emerged as an important tool to profile sperm proteins. High throughput techniques such as LC-MS/MS are able to effectively profile the proteins present in the spermatozoa [18] and identify its role in regulating molecular mechanisms associated with sperm function such as capacitation, hyperactivation, acrosome reaction and fertilization process [19]. Earlier proteomic studies have identified the dysregulation of proteins associated with reproductive function in spermatozoa of men with seminal oxidative stress [20,21]. To our knowledge, this is the first study on the effect of antioxidant treatment on the sperm proteome of idiopathic infertile men. The objectives of this study were, (1) to evaluate the sperm protein profile of idiopathic infertile men pre- and post-antioxidant supplementation, and (2) to validate the proteins associated with the fertilization process using the conventional Western blot technique and correlate these findings with the improved seminogram post-treatment.

## 2. Materials and Methods

### 2.1. Study Design and Subjects

A prospective case controlled study was approved by Institutional Review Board (Permit #15-1006) and carried out at the American Center for Reproductive Medicine, Cleveland Clinic. A written consent was obtained from all the participants enrolled between February 2017 and January 2019.

**Figure 1:**
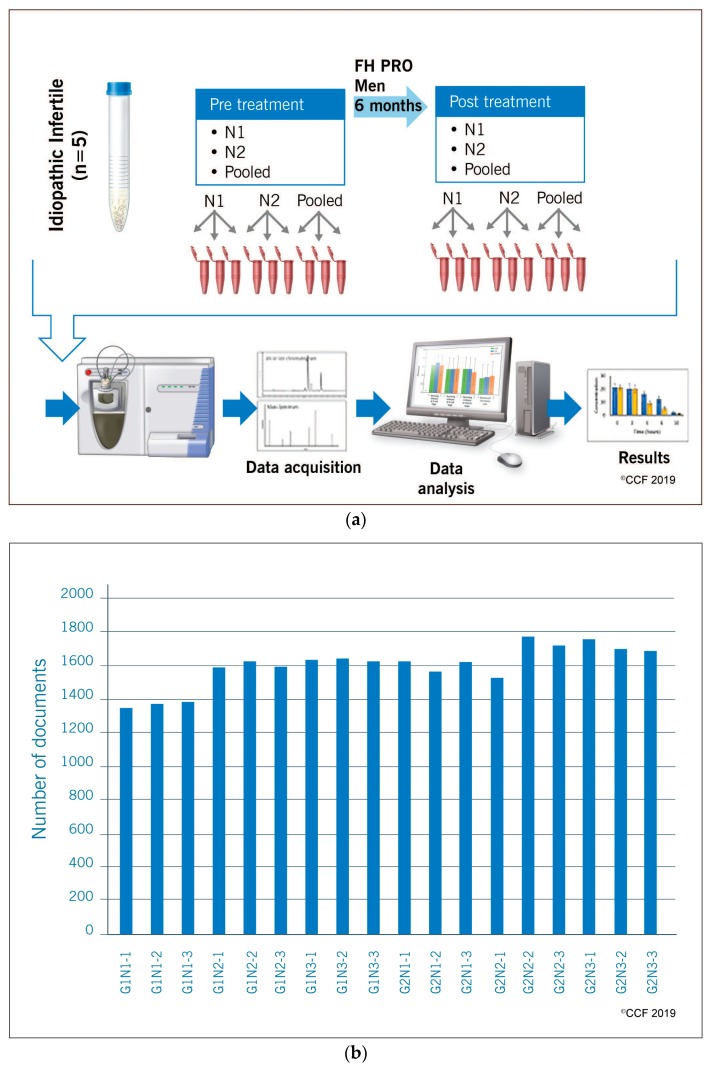
(**a**) A schematic representation of the overall experimental design that was used to identify the proteomic changes associated with antioxidant (‘FH PRO for men’) treatment in idiopathic infertile patients. N1 and N2 represent individual patient samples and the pooled sample is from three different individuals normalized for sperm concentration. Each sample was run in triplicate on one-dimensional SDS-PAGE gels and subjected to in-gel trypsin digestion. The soluble proteins were extracted, reduced, alkylated, and analyzed using liquid chromatography-tandem mass spectrometry. (**b**) Histogram showing homogeneity in the number of proteins identified in individual samples run in triplicate. G1N1 1-3: Gel-1 N1 (replicate 1-3); G1N2 1-3: Gel-1 N2 (replicate 1-3); G1N3 1-3: Gel-1 pooled sample (replicate 1-3); G2N1 1-3: Gel-2 N1 (replicate 1-3); G2N2 1-3: Gel-2 N2 (replicate 1-3); G2N3 1-3: Gel-2 pooled sample (replicate 1-3).

The present study was conducted based on the experimental design, patient selection criteria and antioxidant formulation as reported by Arafa et al. [16] to gain an in-depth understanding of the mechanism of action of the antioxidant formulation against idiopathic infertility. A shotgun proteomic approach was adopted to unravel the mechanistic pathway responsible for improving semen quality. The sample size for this high throughput proteomic study was justified according to Clough et al. whereby to maintain biological variability, semen samples from eight (*n* = 8) idiopathic infertile men were randomly drawn from the cohort [22]. Pilot studies on sperm proteome from our lab revealed no significant intra- and inter-sample variation in the number of sperm proteins detected within a group (Figure 1). To maintain biological variability, we included two individual samples and one pooled sample (from three different individuals) that were run in triplicate to maintain technical variability as per the practice in studies involving high throughput omics technologies. For validation studies by Western blotting, all the eight individual patient samples were used. Patients were provided with ‘FH PRO for Men’ antioxidant capsules (1000 mcg B12, 30 mg zinc, 140 mcg selenium, 350 mg arginine, 2000 mg, 200 mg Co-Q10, 120 mg vitamin C, 200 IU vitamin E) (Fairhaven Health, Bellingham, WA, USA) for a period of six months. Patients were advised not to change their diet and life style during the course of the study. Semen samples were collected pre- and post-antioxidant supplementation. Two semen samples (one week apart) were collected from each patient prior to the start of the antioxidant treatment and another two samples were collected similarly from the same individual after the completion of the antioxidant treatment.

Subjects included in the current study had at least one abnormal semen parameter (i.e., sperm concentration <15 × 10^6^ sperm/mL, motility <40%, sperm vitality <58%, normal sperm morphology <4%), according to World Health Organization (WHO) 2010 guidelines [23]. Patients with recurring fever 90 days prior to semen analysis were excluded from the study. Similarly, men with leukocytospermia (as identified by a positive Endtz test), azoospermia, severe oligozoospermia (<5 million spermatozoa/mL) were excluded from the study. Infertile men with genetic defects and infection of the reproductive tract as diagnosed by andrological examination were excluded in the present study. Study participants with a history of erectile dysfunction, systemic illness, inflammation of the reproductive or urinary tract (orchitis, epididymitis, urethritis, and testicular atrophy), and sexually transmitted diseases were also excluded. Female partners of these infertile men were reported as having normal reproductive health following general gynecological evaluation.

### 2.2. Semen Analysis and Processing of Semen Samples

Semen samples were collected following 2–3 days of sexual abstinence. Samples were allowed to liquefy completely for 20–30 min at 37 °C. Macroscopic semen parameters such as volume, color, pH, and viscosity were measured. For hyperviscous samples, viscosity was broken down mechanically by repeated pipetting. Proteolytic enzymes were not used to treat viscosity as they interfere with proteomic analysis [24]. Semen volume, sperm motility, concentration and morphology were evaluated according to the World Health Organization (WHO) 2010 guidelines [23]. After semen analysis, samples were processed using a 65% single gradient centrifugation. Briefly, liquefied semen was overlaid on the 65% gradient solution and then centrifuged at 300 g for 20 min. The seminal plasma was carefully discarded. The white buffy coat ring (containing round cells, debris, and leukocytes) formed between the seminal plasma and the gradient was also removed and discarded. Finally, the gradient containing the spermatozoa was diluted in phosphate buffered saline (PBS) and subsequently centrifuged at 3000 g for 15 min. The resulting sperm pellet, which was free from any round cells, was stored at −80 °C.

### 2.3. Sperm Protein Extraction and Quantification

Proteomic profiling of sperm samples was performed using liquid chromatography-tandem mass spectrometry before and after antioxidant treatment. The samples were analyzed in compliance with the Minimum Information about a Proteomics Experiment (MIAPE) guidelines of the Human Proteome Organization’s Proteomics Standards Initiative (HUPO-PSI) for reporting proteomics studies [25]. The sperm pellet was washed four times with phosphate buffered saline (PBS; Irvine Scientific, Santa Ana, CA, USA) and centrifuged at 4000× *g* for 10 min, at 4 °C. Radio-immunoprecipitation assay (RIPA; Sigma-Aldrich, St. Louis, MO, USA) buffer supplemented with Protease Inhibitor Cocktail, cOmplete^TM^ ULTRA Tablets, EDTA-free (Roche, Mannheim, Germany) was added to the sperm pellet (100 µL RIPA/10^6^ sperm) and left overnight at 4 °C for cell lysis. The samples were centrifuged at 10,000× *g* for 30 min at 4 °C and the supernatant was then transferred to a new centrifuge tube. Protein quantification in the fractions was performed using the Pierce BCA Protein Assay kit (Thermo Fisher Scientific, Waltham, MA, USA) according to the manufacturer’s instructions.

### 2.4. Liquid Chromatography-Tandem Mass Spectrometry

Firstly, all the samples were mixed with SDS Page buffer. Each sample was run in triplicates. Protein samples were separated on a 1D gel. To elute the proteins from the gel, the bands from each lane were cut and divided into several smaller pieces. The cut gel pieces were washed in H_2_O and then dehydrated in acetonitrile. Further, these gel pieces were reduced and alkylated with dithiothreitol and iodoacetamide respectively. In-gel digestion of the isolated proteins was done using trypsin (5 μL 10 ng/μL in 50 mM ammonium bicarbonate), followed by overnight incubation at room temperature to allow digestion to reach completion. The next day, the peptides were eluted in 50% acetonitrile with 5% formic acid solution in two aliquots of 30 μL each. Both fractions were mixed and evaporated to <10 μL in Speedvac and then resuspended in 1% acetic acid to obtain a final volume of ~30 μL for LC-MS analysis.

All the samples were analyzed using the Finnigan LTQ-Orbitrap Elite hybrid mass spectrometer system. Dionex 15 cm × 75 μm id Acclaim Pepmap C18, 2 μm, 100 Å reversed phase capillary chromatography column was used to perform HPLC. Protein samples at five μL volumes were injected and the peptides eluted from the column by an acetonitrile/0.1% formic acid gradient at a flow rate of 0.25 μL/min were introduced into the source of the mass spectrometer online. The micro-electrospray ion source was operated at 2.5 kV. The digest was analyzed using the data dependent multitask capability of the instrument to acquire a full scan mass spectra to determine the peptide molecular weights and product ion spectra to identify the amino acid sequence of the peptide in successive instrument scans. CID spectra from all the scans were collected and programs such as Mascot and SEQUEST were used analyzed by conducting a search with the human reference sequence databases (http://www.hprd.org/). Next, these search results were uploaded into the program Scaffold (Proteome Software Inc., Portland, OR, USA; version 4.0.6.1). Based on the number of spectral counts, the abundance of each protein in the pool was then classified as either very low, low, medium or high. The normalized spectral abundance factor (NSAF) ratio was subsequently calculated to categorize the expression profile of differentially expressed proteins (DEPs) as underexpressed, overexpressed or unique to one of the groups, as previously described [21]. Different p-values were considered according to the abundance of the proteins: (i) Very Low abundance: spectral count range 1.7–7; *p* ≤ 0.001 and NSAF ratio ≥2.5 for overexpressed, ≤0.4 for underexpressed proteins; (ii) Low abundance: spectral count range 8–19; *p* ≤ 0.01; and NSAF ratio ≥ 2.5 for overexpressed, ≤0.4 for underexpressed proteins; (iii) Medium abundance: spectral count range between 20 and 79; *p* ≤ 0.05 and NSAF ratio ≥2.0 for overexpressed, ≤0.5 for underexpressed proteins; (iv) High abundance: spectral counts > 80; *p* ≤ 0.05 and NSAF ratio ≥1.5 for overexpressed, ≤0.67 for underexpressed proteins.

### 2.5. Bioinformatic Analysis

Functional pathway analysis of the DEPs was carried out using the Ingenuity Pathway Analysis (IPA) (Qiagen, Hilden, Germany) software. This program allowed the evaluation of the top canonical pathways, diseases and bio-functions, and upstream regulators related to the DEPs. Additionally, Metacore™ analysis was carried out to identify the dysregulated pathways in idiopathic infertile men. 

### 2.6. Protein Selection and Validation by Western Blot

To distinguish the expression of sperm proteins in pre- and post-antioxidant treated patients in the setting of fertility potential, sperm proteins related to reproductive function were selected for validation by Western blot (WB). The criteria applied for the selection of DEPs for validation by WB were as follows: (i) proteins involved in the development and function of the reproductive system; (ii) proteins involved in the top canonical pathways; (iii) proteins with well-described functions in literature. Four proteins (NDUFS1, CCT3, PRKAR1A and SPA17) were chosen for validation by WB in individual samples from the pre-antioxidant treatment group. A total of 20 µg of protein per sample was first loaded into a 4%–15% SDS–PAGE for 2 h at 90 V. Next, the resolved protein bands were transferred onto polyvinylidene difluoride (PVDF) membranes, and for each protein analysis, specific primary antibodies were incubated at 4 °C overnight. The membranes were incubated with the secondary antibody at room temperature for 1 h and finally reacted for 5 min with enhanced chemiluminescence (ECL) reagent (GE Healthcare, Marlborough, MA, USA). To detect the chemiluminescence signals, the membranes were exposed to ChemiDoc (ChemiDocTM MP Imaging System, Bio-Rad, Hercules, CA, USA). All the PVDF membranes used for protein identification were exposed to total protein staining. The membranes were briefly washed twice for 10 min in distilled water and stained with total colloidal gold protein stain (Bio-Rad) for 2 h at room temperature by gentle shaking. Stained membranes were washed twice with distilled water for 10 min, and the densitometry image was captured using the colorimetric mode on ChemiDoc (ChemiDocTM MP Imaging System, Bio-Rad).

## 3. Results

### 3.1. Proteomic Profile of Spermatozoa

**Figure 2:**
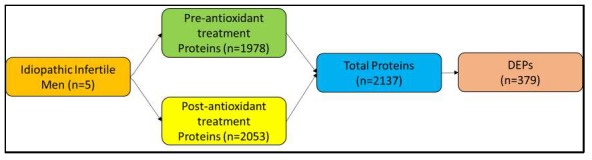
The number of sperm proteins identified in pre-and post-antioxidant treatment groups. Semen sample from 5 patients were used for the analysis in which two were individual sample while the third was a pooled sample from three different individuals.

**Figure 3:**
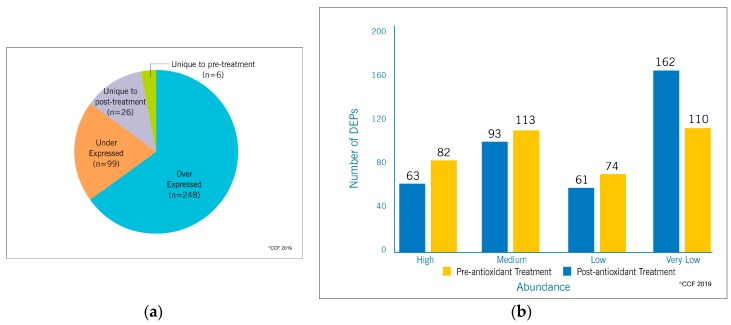
(**a**) The number and (**b**) abundance of differentially expressed proteins identified in spermatozoa of idiopathic infertile patients pre-and post-antioxidant treatment.

Liquid chromatography-tandem mass spectrometry (LC-MS/MS) detected a total of 1978 proteins in the pre-antioxidant treatment group and 2053 proteins in the post-antioxidant treatment group. From a total of 2137 proteins present in both the groups, 379 proteins were identified as DEPs (Figure 2, Supplementary Table S1). Furthermore, 248 and 99 DEPs were over-expressed and under-expressed respectively in the post-antioxidant treatment group. Whereas, six DEPs were unique to pre-antioxidant treatment group and 26 DEPs were unique to post-antioxidant treatment group (Figure 3a). The abundance of the DEPs between both the groups are depicted in Figure 3b.

### 3.2. Functional Annotation and Pathway Analysis

**Figure 4:**
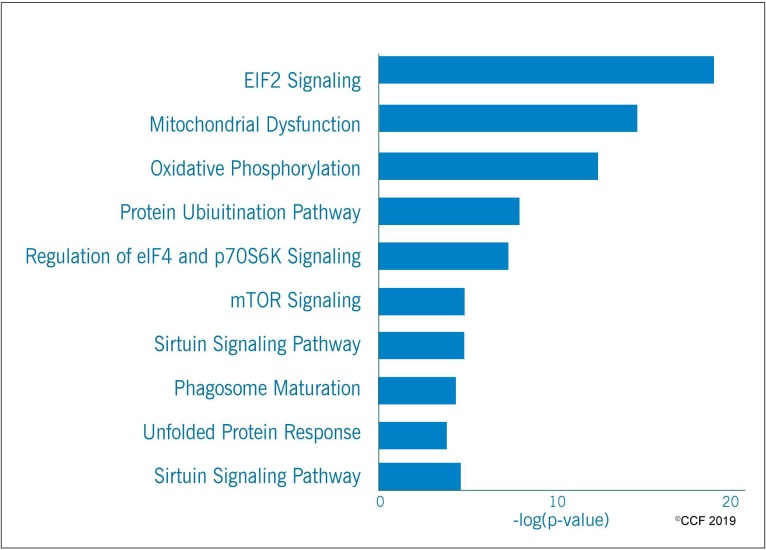
Top ten enriched canonical pathways in idiopathic infertile men post-antioxidant treatment.

**Figure 5:**
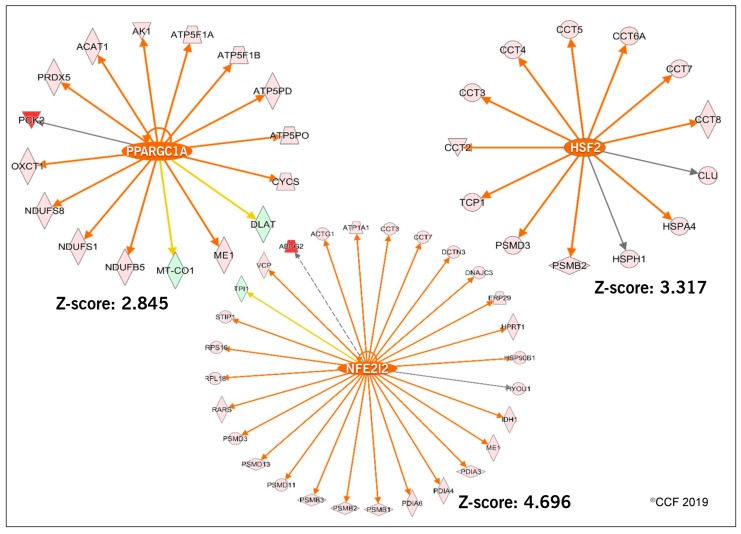
Predicted transcription factors PPARGC1A, NFE2L2 and HSF2 regulating the DEPs in idiopathic infertile men post-antioxidant treatment.

IPA analysis revealed that eIF2 Signaling (endoplasmic reticulum stress) mitochondrial function, oxidative phosphorylation and protein ubiquitination were among the top ten enriched canonical pathways (Figure 4). In addition, the transcriptional factors (TFs) associated with sperm motility and capacitation (PPARGC1A), and free radical scavenging system (NFE2L2 and HSF2) were predicted to be activated in the spermatozoa of idiopathic infertile men post-antioxidant treatment after upstream transcriptional regulator analysis (Figure 5). The majority of the DEPs under the regulation of these TFs were overexpressed. 

**Figure 6:**
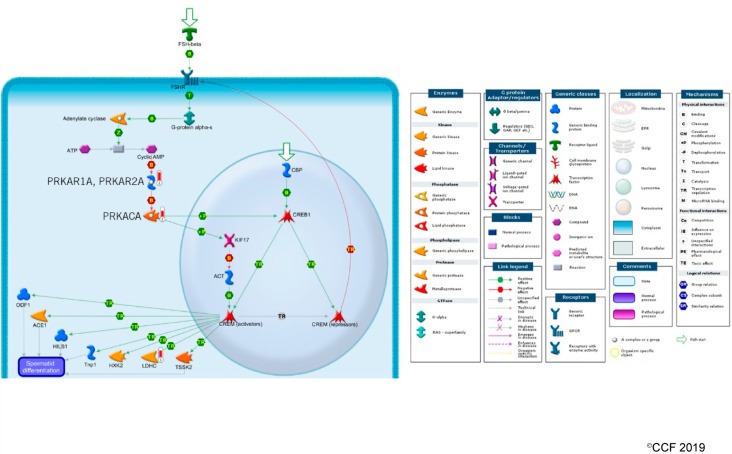
Activation of CREM signaling pathway in spermatozoa of idiopathic infertile patients post-antioxidant treatment.

Transcriptional regulatory network analysis of the DEPs using Metacore™, revealed overexpression of 4 proteins, namely, PRKAR1A and PRKAR2A, PRKACA and LDHC in cAMP responsive element modulator (CREM) signaling in the testis of post-antioxidant treatment group (Figure 6).

### 3.3. Effect of Antioxidant Treatment on Redox Regulation and Reproductive Function

**Figure 7:**
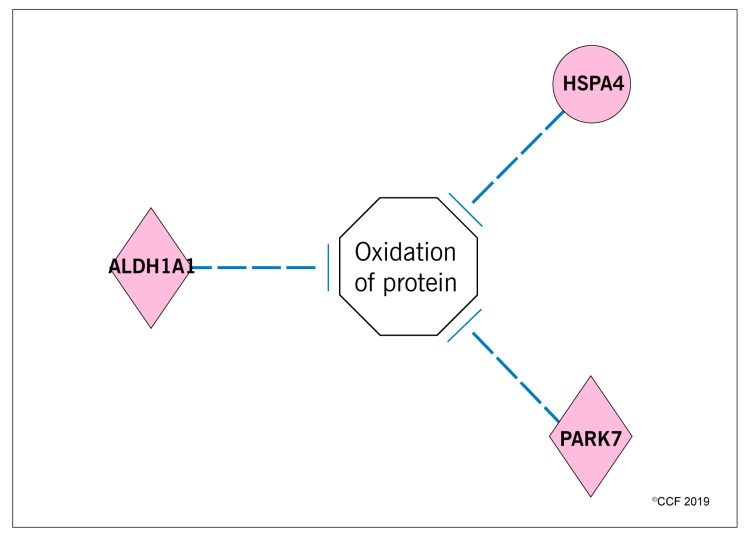
Inhibition of the oxidation of protein pathway in spermatozoa of idiopathic infertile patients post-antioxidant treatment.

Functional analysis identified that the proteins associated with sperm function and fertilization process were altered and the majority of the DEPs were overexpressed in post-antioxidant treatment group (Table 1). IPA analysis identified that the oxidation of protein pathway was inhibited due to overexpression of HSPA4 (4.08 folds), ALDH1A1 (10.32 folds) and PARK7 (2.69 folds) proteins in the post-antioxidant treatment group of idiopathic infertile men (Figure 7). Similarly, the proteins associated with the response to hypoxia and oxidative stress processes such as COX1 (−1.57 folds), GPX4 (−1.40 folds), GSTO2 (−2.59 folds) were underexpressed, whereas PRDX5 (1.47 folds) was overexpressed in the post-antioxidant treatment group. In addition, molecular functions associated with cellular processes such as cell death, necrosis and apoptosis showed a decreased state of activation in spermatozoa of idiopathic infertile men following antioxidant treatment (Table 2). Furthermore, bioinformatics analysis also revealed that the proteins associated with the molecular pathways such as mitochondrial function and stabilization of proteins were overexpressed in post-antioxidant treatment group (Table 3). 

### 3.4. Selection and Validation of Expression Profile of Proteins by Western Blot

**Figure 8:**
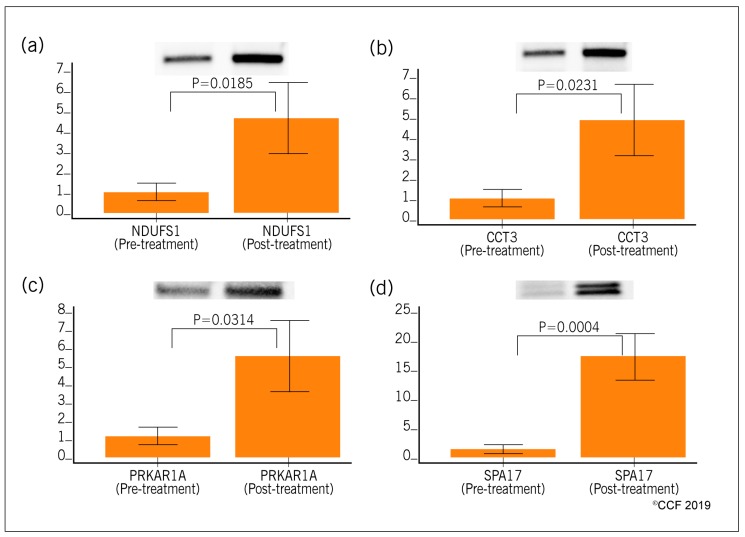
Expression profile of differentially expressed sperm proteins (Western blot) in spermatozoa of idiopathic infertile patients pre- (*n* = 8) and post-antioxidant treatment (*n* = 8). (**a**) NDUFS1, (**b**) CCT3, (**c**) PRKAR1A and (**d**) SPA17.

The proteins were selected for validation based on their association with the vital pathways/processes dysregulated in idiopathic infertile men related to sperm function. Four selected proteins NDUFS1, CCT3, PRKAR1A, and SPA17 were validated using Western blot in both pre- and post-antioxidant treated idiopathic infertile men. Western blot validation revealed overexpression of NDUFS1 (4.75 fold change, *p* = 0.0185), CCT3 (4.93 fold change, *p* = 0.0231), PRKAR1A (5.58 fold change, *p* = 0.0314) and SPA17 (16.85 fold change, *p* = 0.0004) in post-treatment group (Figure 8).

## 4. Discussion

Seminal oxidative stress (OS) results from increased ROS and is considered as a common convergent point of numerous etiologies of male reproductive dysfunction. Endogenous antioxidants maintain the seminal redox balance by scavenging excess ROS [26] and disruption in the intricate balance between these antioxidants and oxidants leads to OS. Therefore, the antioxidant defense system plays a key role in maintaining the seminal redox balance critical for proper male reproductive functions. Oxidative stress is observed in the semen of 30% to 80% of idiopathic infertile men [1]. Therefore, it is pertinent to recommend antioxidant therapy as a treatment modality for these infertile men. In fact, antioxidants given individually or in combination as oral dietary supplements have been shown to significantly improve semen quality in idiopathic interfile men [4,15,27]. Recent clinical trials using a combination antioxidant supplement (‘FH PRO for Men’) in idiopathic infertile men showed significant improvement in overall semen parameters [16,28]. Although antioxidants have been reported to have a beneficial effect on sperm parameters, it is essential to understand the mechanisms/pathways implicated in the observed effects on spermatozoa following antioxidant supplementation. In the current study, we utilized the LC-MS/MS platform to profile the maximum number of sperm proteins in idiopathic infertile men pre- and post-antioxidant treatment (‘FH PRO for Men’) in order to understand the mechanism(s) by which the antioxidant supplement improves semen parameters. Furthermore, the proteins associated with reproductive function and the fertilization process were validated using the WB technique.

Oxidative stress has an adverse effect on the sperm proteome. Earlier proteomics studies showed that proteins associated with spermatogenesis, spermatid development, oxidative stress regulation, protein folding, and protein modifications were dysregulated in infertile men with high levels of seminal ROS [20,29]. In the current study, we observed an overexpression of 248 proteins at the molecular level in idiopathic infertile men treated with ‘FH PRO for Men’ (Figure 2). The overexpressed proteins were found to be under the regulation of TFs PPARGC1A and NFE2L2 (Figure 4). PPARGC1 is a transcription co-activator that mediates the activation of antioxidant defense systems and is mainly involved in the detoxification of the ROS [30]. Similarly, NFE2L2 is a transcriptional activator of cellular anti-oxidant enzymes and was activated in fertile men with high antioxidant defense mechanisms [31]. Since transcription and translation are almost silent in ejaculated spermatozoa, it was hypothesized that overexpression of DEPs under the regulation of PPARGC1 and NFE2L2 are a manifestation of upregulation in the activities of these transcription factors during spermatogenesis. Therefore, activation of PPARGC1A and NFE2L2 in the post-treated group of idiopathic infertile men indicates that ‘FH PRO for Men’ modulates the expression of proteins involved in the antioxidant defense mechanism and has a beneficial effect at the subcellular level to counteract and protect the spermatozoa from oxidative stress. Furthermore, the use of antioxidants had a positive effect on the response to hypoxia and the oxidative stress pathway in sperm of idiopathic infertile men. Testicular hypoxia disturbs the homeostasis and enhances production of ROS [32,33] and has an adverse effect on sperm motility [34]. Proteomic analysis revealed the involvement of DEPs COX1, GPX4, GSTO2 and PRDX5 in hypoxia and oxidative stress pathway. COX1 is a component of the respiratory chain and is involved in the oxidative phosphorylation pathway. COX1 levels are very high in oxidative stress-mediated male infertility conditions [35,36]. Similarly, GSTO2 exhibits a glutathione-dependent thiol transferase activity for xenobiotic metabolism and a high dehydroascorbate reductase activity that may contribute to the recycling of ascorbic acid, thereby exhibiting an indirect antioxidant activity. In mice, this enzyme is involved in nuclear decondensation during fertilization [37,38]. Whereas GPX4 has multiple molecular functions in the spermatozoa such as regulation of mitochondrial membrane potential, chromatin decondensation and detoxification of phospholipid hydroperoxides [39]. Besides, the moonlighting function of GPX4 (as an ROS metabolizing enzyme and structural protein), it is also responsible for the maintenance of mitochondrial stability and consequent male fertility [40]. GPX4 is also required for differential cell death decision in the testis [41] Sharma et al. reported that the levels of GPX4 protein was upregulated in men with high seminal ROS [20]. On the other hand, PRDX5 plays a major role in the antioxidant defense mechanism in the sperm mitochondria [42]. In the current study, the underexpression of COX1, GPX4, GSTO2 in post-antioxidant treated subjects indicates a reduced state of hypoxia and overexpression of PRDX5 suggests an adaptive protective mechanism against oxidative stress. Altogether, the antioxidant formulation present in ‘FH PRO for Men’ effectively changes the expression of the sperm proteins that can counteract the oxidative stress in idiopathic infertile men. 

Mature sperm are transcriptionally and translationally inert and primarily depend on their translated proteins for their biological functions [43]. Any defect in the structure or composition of sperm proteins negatively affects its functions such as capacitation, hyperactivation, acrosome reaction and fertilization [18]. Several proteomic studies have demonstrated that the proteins involved in the vital function pathways or protein modification pathways of spermatozoa were dysregulated in male infertility conditions [20,29,44,45,46,47]. In the antioxidant (‘FH PRO for Men’) treated group, we noticed that the majority of the proteins, specifically chaperones and their associated factors that are involved in stabilization of protein function, were overexpressed (Table 3). In addition, the ‘FH PRO for Men’ formulation was able to increase the expression of HSPA4, ALDH1A1 and PARK7, which in turn may protect the sperm proteome against oxidative damage.

Components of the molecular chaperone complex, TRiC (chaperonin-containing T-complex/TCP1 ring complex) such as CCT2, CCT3, CCT4, CCT5, CCT6A CCT7 and TCP1, that assists in the folding of proteins upon ATP hydrolysis are overexpressed after ‘FH PRO for Men’ treatment suggesting better mediation of sperm-oocyte interaction by proper presentation of ZP receptors [48]. The T-complex protein 1 subunit zeta-2 (CCT6B) is also involved in protein (actin and tubulin) folding mediated by cytoplasmic chaperonin containing TCP-1 and is highly expressed in the testis [49]. The gene products of Bardet–Biedl syndrome (BBS) and McKusick–Kaufman syndrome (MKKS) are involved in vesicular trafficking in cilia or flagella. Disruptions of BBS or MKKS genes result in male infertility owing to the failure of flagellum formation in spermatozoa. The chaperonin-containing T-complex/TCP1-ring complex as part of the BBS/CCT complex may play a significant role in the assembly of BBSome, a complex involved in ciliogenesis regulating transport of vesicles to the cilia. It also plays an important role in the cytoskeletal organization during spermatogenesis. This TRiC complex is also confined to nuclear heterochromatin and associated with nuclear compaction of the spermatozoa. The TRiC complex mediates the folding of WRAP53/TCAB1, thereby regulating telomere maintenance [50]. Telomere length in sperm increases with age. Telomere lengthening in human sperm seemingly arises from the continued action of the enzyme telomerase that is expressed at high levels in spermatogonia. Although sperm maintain a longer average telomere length, sperm telomere length varies among individual males and individual spermatozoa [51]. Variation in sperm telomere length could result from variable activity of telomerase and/or from the effects of oxidative stress, which results in shortened telomeres. In sperm, compromised telomere length may contribute to segregation errors, apoptosis with reduced sperm count, and reduced fertility [52,53]. To corroborate the facts, we observed a significant reduction in expression of proteins involved in apoptosis pathway after antioxidant treatment (Table 3). This clearly indicates that the ‘FH PRO for Men’ has a vital property to retain the expression of the proteins associated with spermatogenesis, ciliogenesis, sperm cell maturation and protein stabilization, and protects it from the oxidation process, thereby improving the semen quality in idiopathic infertile men. 

Organelles that are intact and functional reflect the fertility potential of the spermatozoa, of which mitochondria, the powerhouse of sperm, deserves special mention. Mitochondrial dysfunction is directly associated with male infertility issues [54]. ROS produced by mitochondria has a negative impact on sperm plasma membrane and its sperm DNA integrity [5,55]. Mitochondrial dysfunction also leads to cell death either by activating necrosis or apoptosis pathways [56,57]. Antioxidant treatment in idiopathic infertile men that resulted in deactivation or inhibition of cellular pathways such as cell death, necrosis and apoptosis were found to be deactivated or inhibited (Table 2). Earlier proteomic studies have reported the alteration of mitochondrial proteins in oxidative stress-mediated male infertility [45,58,59]. Underexpression of the mitochondrial proteins were especially prominent in unexplained male infertility cases [44]. In our proteomics study, we have observed a state of underexpressed mitochondrial proteins in spermatozoa of idiopathic male infertility cases and their expression increased significantly after oral intake of ‘FH PRO for Men’ for a period of six months (Table 2). Validation of the mitochondrial protein NDUFS1 using Western blot in both pre- and post-antioxidant treated samples supports our proteomic findings. NDUFS1 is an inner mitochondrial membrane protein involved in the transfer of electrons in the oxidative phosphorylation process. Overexpression of NDUFS1 suggests that the current antioxidant formulation has the capacity to augment the activity of the mitochondrial complex I, one of the principal sites of superoxide generation [60,61]. In addition, we have noticed that NDUFS1 is under the regulation of activated transcriptional factor PPARGC1A involved in maintaining the redox balance. Hence, NDUFS1 may serve as potential diagnostic biomarker to access the mitochondrial function in idiopathic infertile men under the antioxidant treatment. 

The fertility potential of men is directly related to their semen and/or sperm quality. Physiologically normal spermatogenesis ensures the production of matured sperm that are able to bind and fertilize the ova [62]. Defective spermatogenesis is a common cause of male infertility [63]. Kothandaraman et al., 2016 reported that 135 genes/molecules were associated with the spermatogenesis process in idiopathic male infertility cases [64]. Several other investigators have also demonstrated the dysregulation of proteins associated with spermatogenesis in male infertility cases [64,65,66]. Recently, we have demonstrated that proteins related to spermatogenesis were underexpressed in men with high seminal ROS levels, suggesting the disruption of the spermatogenesis process that resulted in ROS-induced infertility [29]. In the present study, antioxidant treatment resulted in the increased expression of proteins associated with spermatogenesis, maturation of sperm, sperm-binding function and fertilization process (Table 3). Validation of SPA17 and CCT3 proteins using Western blot were in agreement with the proteomic findings. SPA17 plays an important role in sperm motility and binds to the extracellular matrix of the oocyte [67,68]. It was underexpressed in men with primary or secondary infertility [69] and hence underexpression of SPA17 may affect sperm capacitation and sperm–oocyte binding in idiopathic infertile men. In the present study, validation of underexpression of SPA17 in idiopathic infertile men confirms the compromised spermatogenesis and fertilization processes in idiopathic infertile men. Whereas, increase in the expression of SPA17 in these same patients following antioxidant treatment indicates the role of antioxidants in the restoration of physiological spermatogenesis in idiopathic infertile men. Furthermore, we have noticed the upregulation of PRKAR1A, PRKAR2A, PRKACA, and LDHC in the CREM signaling pathway (Figure 5) post-antioxidant treatment. PRKAC is the catalytic subunit, while PRKAR1A, PRKAR2A are regulatory subunits of the cAMP-dependent protein kinases (PKA). The catalytic activity and subcellular localization of PKA depends on the regulatory subunits. Both phosphorylation and oxidation regulate PKA activity. The free C-subunit can be inactivated by oxidation of Cys199. This cysteine is competent to form an internal disulfide with Cys in the C terminus or a mixed disulfide with glutathione, effectively inactivating the enzyme a redox-sensitive manner [70]. Therefore, shifting of the oxidative predominance in idiopathic infertility patients to a reducing one by ‘FH Pro for Men’ antioxidant may improve PKA activity, thereby, enhance cellular signaling. Additionally, heterozygotes for PRKAR1A are infertile and had lower sperm count with morphologically aberrant spermatozoa [71]. It will not be out of context to mention here that, defective expression of CREM results in an arrest of spermatid differentiation and apoptosis [72,73,74]. CREM element was deactivated in men with high seminal ROS [20]. 

## 5. Conclusions

In current medical practice, antioxidant therapy is widely used in the management of oxidative stress-induced male infertility and idiopathic male infertility. However, its action at subcellular levels remain unclear. This novel proteomic study helps to decipher the role of antioxidants in activating the antioxidant defense mechanism at a subcellular level along with its beneficial effect on sperm proteins essential for the fertilization process. Our proteomic findings suggest that sperm proteins NDUFS1, CCT3, PRKAR1A and SPA17 can serve as potential biomarkers to monitor the effect of antioxidant therapy in men with idiopathic infertility. Furthermore, these findings will serve to guide physicians in determining better treatment options for the management of male infertility. A limitation of this study is that the data on pregnancy outcome in these patients is not available. Future longitudinal studies on pregnancy and live birth rates will further strengthen our findings.

## Figures and Tables

**Table 1:** Overexpressed proteins in the process related sperm function and fertilization in spermatozoa of idiopathic infertile men post-antioxidant treatment.

Function	Proteins (Expression Fold Change)
Binding of sperm	CCT2 (2.23), CCT3 (1.93), CCT4 (2.38), CCT5 (2.49), CCT6A (2.92), CCT7 (1.94), CCT8 (2.28), SPA17 (8.81), TCP1 (2.30)
Fertilization	LDHC (3.12), PARK7 (2.69), PRKACA (2.53), SPA17 (8.81), SPESP1 (3.89)
Maturation of sperm	CLU (2.36), TPP2 (5.30)
Spermatogenesis	CCT6B (2.64), DPCD (2.72), HSPA4 (4.08), HSPA4L (3.80), IMMP2L (3.03), NPEPPS (4.16), PDILT (3.69), SPA17 (8.81), TCP1 (2.30)

**Table 2:** Activation state of the cellular pathways in spermatozoa of idiopathic infertile men post-antioxidant treatment.

Cellular Pathways	# of DEPs	Activation State	Z-Score
Cell death	142	Decreased	−4.786
Necrosis	124	Decreased	−4.877
Apoptosis	89	Decreased	−3.748

**Table 3:** Overexpressed sperm proteins associated with molecular pathways in post-antioxidant treatment group.

Function/Pathways	Proteins (Expression Fold Change)
Mitochondrial function	ACO2 (1.51), ATP5F1A (1.81), ATP5F1B (2.76), ATP5F1C (2.12), ATP5F1D (8.05), ATP5MG (2.57), ATP5PD (2.82), ATP5PO (2.19), CYCS (2.89), HSD17B10 (1.70), NDUFA6 (2.70), NDUFA9 (2.49), NDUFA13 (3.30), NDUFB5 (2.99), NDUFS1 (1.83), NDUFS8 (3.70), PARK7 (2.69), PRDX5 (2.78), UQCRB (2.04)
Stabilization of proteins	APOA1 (4.88), CALR (1.73), CCT2 (2.23), CCT3 (1.93), CCT4 (2.38), CCT5 (2.49), CCT6A (2.92), CCT7 (1.94), CCT8 (2.28), CLU (2.36), HSPD1 (3.26), PARK7 (2.69), PHB (3.82), PHB2 (3.32), RPL11 (2.66), RPL5 (2.59), RPS7 (6.00), STX12 (2.84), TCP1 (2.30)

## Supplementary Materials

The following are available online at https://www.mdpi.com/2076-3921/8/10/488/s1, Table S1: Differentially expressed proteins in spermatozoa of idiopathic infertile patients pre- and post-antioxidant treatment.

## References

[1] Agarwal A., Parekh N., Panner Selvam M.K., Henkel R., Shah R., Homa S.T., Ramasamy R., Ko E., Tremellen K., Esteves S. (2019). Male Oxidative Stress Infertility (MOSI): Proposed Terminology and Clinical Practice Guidelines for Management of Idiopathic Male Infertility. World J. Mens. Health.

[2] Bui A.D., Sharma R., Henkel R., Agarwal A. (2018). Reactive oxygen species impact on sperm DNA and its role in male infertility. Andrologia.

[3] Aitken R.J., De Iuliis G.N., Drevet J.R., Henkel R., Samanta L., Agarwal A. (2019). Chapter 2.2—Role of Oxidative Stress in the Etiology of Male Infertility and the Potential Therapeutic Value of Antioxidants. Oxidants, Antioxidants and Impact of the Oxidative Status in Male Reproduction.

[4] Tremellen K. (2008). Oxidative stress and male infertility—A clinical perspective. Hum. Reprod. Update.

[5] Wagner H., Cheng J.W., Ko E.Y. (2018). Role of reactive oxygen species in male infertility: An updated review of literature. Arab J. Urol..

[6] Agarwal A., Sharma R., Roychoudhury S., Du Plessis S., Sabanegh E. (2016). MiOXSYS: A novel method of measuring oxidation reduction potential in semen and seminal plasma. Fertil. Steril..

[7] Agarwal A., Wang S.M. (2017). Clinical Relevance of Oxidation-Reduction Potential in the Evaluation of Male Infertility. Urology.

[8] Agarwal A., Saleh R.A., Bedaiwy M.A. (2003). Role of reactive oxygen species in the pathophysiology of human reproduction. Fertil. Steril..

[9] Aitken R.J., Koppers A.J. (2011). Apoptosis and DNA damage in human spermatozoa. Asian J. Androl..

[10] Dorostghoal M., Kazeminejad S.R., Shahbazian N., Pourmehdi M., Jabbari A. (2017). Oxidative stress status and sperm DNA fragmentation in fertile and infertile men. Andrologia.

[11] Balercia G., Regoli F., Armeni T., Koverech A., Mantero F., Boscaro M. (2005). Placebo-controlled double-blind randomized trial on the use of l-carnitine, l-acetylcarnitine, or combined l-carnitine and l-acetylcarnitine in men with idiopathic asthenozoospermia. Fertil. Steril..

[12] Safarinejad M.R., Safarinejad S., Shafiei N., Safarinejad S. (2012). Effects of the Reduced Form of Coenzyme Q_10_ (Ubiquinol) on Semen Parameters in Men with Idiopathic Infertility: A Double-Blind, Placebo Controlled, Randomized Study. J. Urol..

[13] ElSheikh M.G., Hosny M.B., Elshenoufy A., Elghamrawi H., Fayad A., Abdelrahman S. (2015). Combination of vitamin E and clomiphene citrate in treating patients with idiopathic oligoasthenozoospermia: A prospective, randomized trial. Andrology.

[14] Majzoub A., Agarwal A., Esteves S.C. (2017). Antioxidants for elevated sperm DNA fragmentation: A mini review. Transl. Androl. Urol..

[15] Micic S., Lalic N., Djordjevic D., Bojanic N., Bogavac-Stanojevic N., Busetto G.M., Virmani A., Agarwal A. (2019). Double-blind, randomised, placebo-controlled trial on the effect of L-carnitine and L-acetylcarnitine on sperm parameters in men with idiopathic oligoasthenozoospermia. Andrologia.

[16] Arafa M., Agarwal A., Majzoub A., Khalafalla K., Alsaid S., Elbardisi H. (2019). Efficacy of antioxidant supplementation on conventional and advanced sperm function tests in patients with idiopathic male infertility. Fertil. Steril..

[17] Gharagozloo P., Gutierrez-Adan A., Champroux A., Noblanc A., Kocer A., Calle A., Perez-Cerezales S., Pericuesta E., Polhemus A., Moazamian A. (2016). A novel antioxidant formulation designed to treat male infertility associated with oxidative stress: Promising preclinical evidence from animal models. Hum. Reprod..

[18] Panner Selvam M.K., Agarwal A. (2018). Update on the proteomics of male infertility: A systematic review. Arab J. Urol..

[19] Xu W., Hu H., Wang Z., Chen X., Yang F., Zhu Z., Fang P., Dai J., Wang L., Shi H. (2012). Proteomic characteristics of spermatozoa in normozoospermic patients with infertility. J. Proteom..

[20] Sharma R., Agarwal A., Mohanty G., Hamada A.J., Gopalan B., Willard B., Yadav S., du Plessis S. (2013). Proteomic analysis of human spermatozoa proteins with oxidative stress. Reprod. Biol. Endocrinol. RBE.

[21] Agarwal A., Ayaz A., Samanta L., Sharma R., Assidi M., Abuzenadah A.M., Sabanegh E. (2015). Comparative proteomic network signatures in seminal plasma of infertile men as a function of reactive oxygen species. Clin. Proteom..

[22] Clough T., Thaminy S., Ragg S., Aebersold R., Vitek O. (2012). Statistical protein quantification and significance analysis in label-free LC-MS experiments with complex designs. BMC Bioinform..

[23] WHO (2010). WHO Laboratory Manual for the Examination and Processing of Human Semen.

[24] Panner Selvam M.K., Agarwal A., Sharma R., Samanta L. (2018). Treatment of semen samples with α-chymotrypsin alters the expression pattern of sperm functional proteins—A pilot study. Andrology.

[25] Martínez-Bartolomé S., Deutsch E.W., Binz P.-A., Jones A.R., Eisenacher M., Mayer G., Campos A., Canals F., Bech-Serra J.-J., Carrascal M. (2013). Guidelines for reporting quantitative mass spectrometry based experiments in proteomics. J. Proteom..

[26] Agarwal A., Sekhon L.H. (2010). The role of antioxidant therapy in the treatment of male infertility. Hum. Fertil..

[27] Balercia G., Buldreghini E., Vignini A., Tiano L., Paggi F., Amoroso S., Ricciardo-Lamonica G., Boscaro M., Lenzi A., Littarru G. (2009). Coenzyme Q10 treatment in infertile men with idiopathic asthenozoospermia: A placebo-controlled, double-blind randomized trial. Fertil. Steril..

[28] Khalafalla K., Arafa M., Majzoub A., Elbardisi H., Alsaid S., Agarwal A. (2019). Antioxidant combination therapy: A new hope for oligoathenoteratospermic patients. Fertil. Steril..

[29] Ayaz A., Agarwal A., Sharma R., Arafa M., Elbardisi H., Cui Z. (2015). Impact of precise modulation of reactive oxygen species levels on spermatozoa proteins in infertile men. Clin Proteom..

[30] Guvvala P.R., Ravindra J.P., Selvaraju S., Arangasamy A., Venkata K.M. (2019). Ellagic and ferulic acids protect arsenic-induced male reproductive toxicity via regulating Nfe2l2, Ppargc1a and StAR expressions in testis. Toxicology.

[31] Dias T.R., Samanta L., Agarwal A., Pushparaj P.N., Panner Selvam M.K., Sharma R. (2019). Proteomic Signatures Reveal Differences in Stress Response, Antioxidant Defense and Proteasomal Activity in Fertile Men with High Seminal ROS Levels. Int. J. Mol. Sci..

[32] Said T.M., Grunewald S., Paasch U., Rasch M., Agarwal A., Glander H.-J. (2005). Effects of magnetic-activated cell sorting on sperm motility and cryosurvival rates. Fertil. Steril..

[33] Dutta S., Majzoub A., Agarwal A. (2019). Oxidative stress and sperm function: A systematic review on evaluation and management. Arab J. Urol..

[34] Verratti V., Di Giulio C., D’Angeli A., Tafuri A., Francavilla S., Pelliccione F. (2016). Sperm forward motility is negatively affected by short-term exposure to altitude hypoxia. Andrologia.

[35] Mostafa T., Rashed L., Taymour M. (2016). Seminal cyclooxygenase relationship with oxidative stress in infertile oligoasthenoteratozoospermic men with varicocele. Andrologia.

[36] Perrotta I., Santoro M., Guido C., Avena P., Tripepi S., De Amicis F., Gervasi M.C., Aquila S. (2012). Expression of cyclooxygenase-1 (COX-1) and COX-2 in human male gametes from normal patients, and those with varicocele and diabetes: A potential molecular marker for diagnosing male infertility disorders. J. Anat..

[37] Hamilton L.E., Suzuki J., Aguila L., Meinsohn M.-C., Smith O.E., Protopapas N., Xu W., Sutovsky P., Oko R. (2019). Sperm-borne glutathione-S-transferase omega 2 accelerates the nuclear decondensation of spermatozoa during fertilization in mice. Biol. Reprod..

[38] Schmuck E.M., Board P.G., Whitbread A.K., Tetlow N., Cavanaugh J.A., Blackburn A.C., Masoumi A. (2005). Characterization of the monomethylarsonate reductase and dehydroascorbate reductase activities of Omega class glutathione transferase variants: Implications for arsenic metabolism and the age-at-onset of Alzheimer’s and Parkinson’s diseases. Pharm. Genom..

[39] Fujii J., Imai H. (2014). Redox reactions in mammalian spermatogenesis and the potential targets of reactive oxygen species under oxidative stress. Spermatogenesis.

[40] Brutsch S.H., Rademacher M., Roth S.R., Muller K., Eder S., Viertel D., Franz C., Kuhn H., Borchert A. (2016). Male Subfertility Induced by Heterozygous Expression of Catalytically Inactive Glutathione Peroxidase 4 Is Rescued in Vivo by Systemic Inactivation of the Alox15 Gene. J. Biol. Chem..

[41] Bromfield E.G., Walters J.L.H., Cafe S.L., Bernstein I.R., Stanger S.J., Anderson A.L., Aitken R.J., McLaughlin E.A., Dun M.D., Gadella B.M. (2019). Differential cell death decisions in the testis: Evidence for an exclusive window of ferroptosis in round spermatids. Mol. Hum. Reprod..

[42] O’Flaherty C., de Souza A.R. (2011). Hydrogen peroxide modifies human sperm peroxiredoxins in a dose-dependent manner. Biol. Reprod..

[43] Baker M.A., Nixon B., Naumovski N., Aitken R.J. (2012). Proteomic insights into the maturation and capacitation of mammalian spermatozoa. Syst. Biol. Reprod. Med..

[44] Panner Selvam M.K., Agarwal A., Pushparaj P.N., Baskaran S., Bendou H. (2019). Sperm Proteome Analysis and Identification of Fertility-Associated Biomarkers in Unexplained Male Infertility. Genes.

[45] Samanta L., Agarwal A., Swain N., Sharma R., Gopalan B., Esteves S.C., Durairajanayagam D., Sabanegh E. (2018). Proteomic Signatures of Sperm Mitochondria in Varicocele: Clinical Use as Biomarkers of Varicocele Associated Infertility. J. Urol..

[46] Cao X., Cui Y., Zhang X., Lou J., Zhou J., Bei H., Wei R. (2018). Proteomic profile of human spermatozoa in healthy and asthenozoospermic individuals. Reprod. Biol. Endocrinol..

[47] Dad B.R., Li-Jun H. (2017). Posttranslational Modifications in Spermatozoa and Effects on Male Fertility and Sperm Viability. OMICS A J. Integr. Biol..

[48] Dun M.D., Smith N.D., Baker M.A., Lin M., Aitken R.J., Nixon B. (2011). The Chaperonin Containing TCP1 Complex (CCT/TRiC) Is Involved in Mediating Sperm-Oocyte Interaction. J. Biol. Chem..

[49] Kubota H., Hynes G.M., Kerr S.M., Willison K.R. (1997). Tissue-specific subunit of the mouse cytosolic chaperonin-containing TCP-1 1. FEBS Lett..

[50] Freund A., Zhong F.L., Venteicher A.S., Meng Z., Veenstra T.D., Frydman J., Artandi S.E. (2014). Proteostatic control of telomerase function through TRiC-mediated folding of TCAB1. Cell.

[51] Baird D.M., Britt-Compton B., Rowson J., Amso N.N., Gregory L., Kipling D. (2006). Telomere instability in the male germline. Hum. Mol. Genet..

[52] Thilagavathi J., Venkatesh S., Dada R. (2013). Telomere length in reproduction. Andrologia.

[53] Ioannou D., Griffin D.K. (2011). Male fertility, chromosome abnormalities, and nuclear organization. Cytogenet. Genome Res..

[54] Nakada K., Sato A., Yoshida K., Morita T., Tanaka H., Inoue S.-I., Yonekawa H., Hayashi J.-I. (2006). Mitochondria-related male infertility. Proc. Natl. Acad. Sci. USA.

[55] Wang X., Sharma R.K., Gupta A., George V., Thomas A.J., Falcone T., Agarwal A. (2003). Alterations in mitochondria membrane potential and oxidative stress in infertile men: A prospective observational study. Fertil. Steril..

[56] Lemasters J.J., Qian T., Bradham C.A., Brenner D.A., Cascio W.E., Trost L.C., Nishimura Y., Nieminen A.-L., Herman B. (1999). Mitochondrial Dysfunction in the Pathogenesis of Necrotic and Apoptotic Cell Death. J. Bioenerg. Biomembr..

[57] Wang C., Youle R.J. (2009). The role of mitochondria in apoptosis. Annu. Rev. Genet..

[58] Nowicka-Bauer K., Lepczynski A., Ozgo M., Kamieniczna M., Fraczek M., Stanski L., Olszewska M., Malcher A., Skrzypczak W., Kurpisz M. (2018). Sperm mitochondrial dysfunction and oxidative stress as possible reasons for isolated asthenozoospermia. J. Physiol. Pharm..

[59] Agarwal A., Sharma R., Samanta L., Durairajanayagam D., Sabanegh E. (2016). Proteomic signatures of infertile men with clinical varicocele and their validation studies reveal mitochondrial dysfunction leading to infertility. Asian J. Androl..

[60] Kato T., Hirano A., Manaka H., Sasaki H., Katagiri T., Kawanami T., Shikama Y., Seino T., Sasaki H. (1991). Calcitonin gene-related peptide immunoreactivity in familial amyotrophic lateral sclerosis. Neurosci. Lett..

[61] Hernansanz-Agustin P., Ramos E., Navarro E., Parada E., Sanchez-Lopez N., Pelaez-Aguado L., Cabrera-Garcia J.D., Tello D., Buendia I., Marina A. (2017). Mitochondrial complex I deactivation is related to superoxide production in acute hypoxia. Redox Biol..

[62] Durairajanayagam D., Rengan A.K., Sharma R.K., Agarwal A., Schattman G.L., Esteves S.C., Agarwal A. (2015). Sperm Biology from Production to Ejaculation. Unexplained Infertility: Pathophysiology, Evaluation and Treatment.

[63] Rajender S., Avery K., Agarwal A. (2011). Epigenetics, spermatogenesis and male infertility. Mutat. Res. Rev. Mutat. Res..

[64] Kothandaraman N., Agarwal A., Abu-Elmagd M., Al-Qahtani M.H. (2016). Pathogenic landscape of idiopathic male infertility: New insight towards its regulatory networks. NPJ Genom. Med..

[65] Bracke A., Peeters K., Punjabi U., Hoogewijs D., Dewilde S. (2018). A search for molecular mechanisms underlying male idiopathic infertility. Reprod. Biomed. Online.

[66] Huang X.Y., Sha J.H. (2011). Proteomics of spermatogenesis: From protein lists to understanding the regulation of male fertility and infertility. Asian J. Androl..

[67] O’rand M.G., Richardson R.T., Yamasaki N. (1995). Expression of the rabbit sperm protein Sp17 in cos cells and interaction of recombinant Sp17 with the rabbit zona pellucida. Mol. Reprod. Dev..

[68] Grizzi F., Chiriva-Internati M., Franceschini B., Hermonat P.L., Soda G., Lim S.H., Dioguardi N. (2003). Immunolocalization of Sperm Protein 17 in Human Testis and Ejaculated Spermatozoa. J. Histochem. Cytochem..

[69] Intasqui P., Agarwal A., Sharma R., Samanta L., Bertolla R.P. (2018). Towards the identification of reliable sperm biomarkers for male infertility: A sperm proteomic approach. Andrologia.

[70] Humphries K.M., Deal M.S., Taylor S.S. (2005). Enhanced dephosphorylation of cAMP-dependent protein kinase by oxidation and thiol modification. J. Biol. Chem..

[71] Burton K.A., McDermott D.A., Wilkes D., Poulsen M.N., Nolan M.A., Goldstein M., Basson C.T., McKnight G.S. (2006). Haploinsufficiency at the protein kinase A RI alpha gene locus leads to fertility defects in male mice and men. Mol. Endocrinol..

[72] Hogeveen K.N., Sassone-Corsi P. (2006). Regulation of gene expression in post-meiotic male germ cells: CREM-signalling pathways and male fertility. Hum. Fertil..

[73] Monaco L., Kotaja N., Fienga G., Hogeveen K., Kolthur U.S., Kimmins S., Brancorsini S., Macho B., Sassone-Corsi P. (2004). Specialized rules of gene transcription in male germ cells: The CREM paradigm. Int. J. Androl..

[74] Weinbauer G.F., Behr R., Bergmann M., Nieschlag E. (1998). Testicular cAMP responsive element modulator (CREM) protein is expressed in round spermatids but is absent or reduced in men with round spermatid maturation arrest. Mol. Hum. Reprod..

